# Sustainable pretreatment method of lignocellulosic depolymerization for enhanced ruminant productivity using laccase protein immobilized agarose beads

**DOI:** 10.1038/s41598-024-76278-0

**Published:** 2024-10-27

**Authors:** Vidya Pradeep Kumar, Manpal Sridhar

**Affiliations:** ICAR-National Institute of Animal Nutrition and Physiology, Adugodi, Bangalore, Karnataka 560030 India

**Keywords:** Laccase, Immobilization, Crop residues, Ruminants, Nutrition, Biological techniques, Biophysics, Chemical biology

## Abstract

Laccase, the selectively lignin degrader, vital to the initiation of lignocellulosic deconstruction was immobilized onto activated agarose beads to increase its reuse potential. Laccase cross-linked beads (~ 3.42 mm) recorded a specific activity of 23 Umg^− 1^, retaining about 80.43% enzyme activity after 45 days of storage. The immobilization yield and efficiency were 89% and 97% respectively. The equilibrium data fitted the Freundlich equation (R^2^ = 0.9987) demonstrating multilayer adsorption and the presence of Cu, Fe, and S in the elemental analysis of immobilized beads established effective binding between activated agarose beads and the laccase protein. Characterization studies of the immobilized laccase-treated crop residues revealed significant differences in the lignin polymer after each treatment cycle. An increase in digestibility of 26.21% and 7.62% was observed in paddy and finger millet-treated straws respectively, over the controls corroborating efficient lignin depolymerization. The propitious performance of laccase beads authenticated in the batch enzymatic reactor to treat crop residues paves headway as a sustainable green technology in the deconstruction of crop residues for use as ruminant feed, augmenting productivity.

**Supplementary Information:**

The online version contains supplementary material available at 10.1038/s41598-024-76278-0.

## Introduction

The lignocellulosic biomass perfectly fits in as a ruminant feedstock for the Indian sub-continent, with its enormous renewable energy potential. Lignocellulosic biomasses such as energy crops (e.g., perennial grasses), agricultural residues (e.g., wheat straw, corn stover, and sugarcane bagasse), forest materials (mainly woody materials), and industrial and municipal wastes are currently the main feedstocks in the bio-based economy^1^. With an annual production of around 170 billion metric tonnes^2^, biomass forms the only renewable organic carbon resource in nature. According to the projected IRENA’s global renewable energy roadmap, Remap 2030, the global biomass demand in the United States, China, India, Brazil, and Indonesia together account for 56% of the total^3^, out of which 75% agricultural field residues generated in India, is utilized as fodder and for other purposes in agriculture and households such as mulching, composting, fuel, thatch, etc^4^.

However, the energy-rich lignocellulosic biomass is underutilized by the livestock, because of a recalcitrant lignin barrier, making a pre-treatment step exclusively mandatory. The physicochemical methods to tackle recalcitrant lignin, are taking the back seat, as these methods in addition to lignin depolymerization, contribute to greenhouse gas emissions by generating phenolics and other toxic compounds^5^. The biological methods on the other hand, especially employing fungi eliminate the production of inhibitors (furfural, phenolic aldehydes) which are otherwise generated using other lignocellulosic pretreatments^6^. The demand for the use and reuse of white rot fungal ligninolytic enzymes laccases is increasing rapidly and the development of new and sustainable methods in depolymerization of lignin employing laccase will be offering affordable and environmentally friendly solutions^7^. Lignin depolymerization using laccase begins with a loss of an electron from the phenolic hydroxyl groups of lignin-producing phenoxy radicals which immediately re-organizes giving α-carbon oxidation or Cα-Cβ bond cleavage of alkyl side chains of the polymer to produce low molecular weight polymeric products^8^ utilizing molecular oxygen as an oxidant and also oxidize phenolic rings to phenoxy radicals^9^ classifying laccase-based approaches as ‘green-chemistry’ approaches^10^.

Repeated use of a biocatalyst is proportional to its stability and the stability of water-soluble enzymes in solution is extremely low^11^ because of higher consumption, shorter half-life, difficulty in separation, recovery, and recycling, etc. Stability can be achieved through a plethora of options like protein oligomerization^12^; cross-linking^13^; protein engineering^14^, and enzyme immobilization^15^. Immobilization offers advantages in terms of operational stability, recovery, and reuse advocating the circular economy model endorsing the “reduce-reuse-recycle” approach^16^. Earlier immobilization of laccases has been extensively studied for various applications, with most of the studies being directed toward applications in dye decolorization^17^. Previously *Trametes versicolor* laccase was immobilized onto Cu-alginate beads by entrapment method to degrade bisphenol A in an aqueous medium demonstrating higher storage stability with greater dye degradation potential^18^. Delignified spent grain was employed for immobilization with laccase obtained from *Trametes versicolor* using the H_2_SO_4_/NaOH digestion^19^ for the removal of synthetic dyes from industrial effluents. Alginate gelatine mixed with immobilized laccase was used for the decolorating of crystal violet^20^. Mixed polymeric microsphere immobilized *Trametes versicolor* laccase was used for methyl red decomposition^21^. Shanmugam et al.^22^ immobilized *Trichoderma asperellum* laccase onto Fe_3_O_4_-SiO_2_-chitosan nano support for biohydrogen production through sweet sorghum stover delignification.

Several research studies^23–25^ conducted to use laccases for delignification of crop residues are targeted for biofuel or bioethanol conversions, making use of the laccase-based oxidation reactions as a preliminary step to tackle the recalcitrant lignin. Very limited studies are available in the literature^26–28^ on the use of laccase de-lignified crop residues as an energy source in ruminant feed. A laccase treatment system mediated by 1-hydroxybenzoic triazole was employed to modify and degrade lignin to enhance the enzymatic saccharification of wheat straw^29^. Earlier studies using laccases have shown encouraging results of lignin depolymerization in crop residues for animal feed^26^. The present study is an attempt to overcome the challenges and limitations associated with the use of soluble enzymes in animal feed conversions, by finding solutions for repeated use of the biocatalyst through an immobilization process: for producing a more stable laccase enzyme through covalent cross-linking on activated agarose beads (AAB); by treatment of crop residues using immobilized laccase beads (IB) in a working prototype of a batch enzymatic reactor for effective lignin depolymerization and through salvaging the IB for multiple reaction cycles establishing a cost-effective recovery process.

## Materials and methods

### Chemicals

Unless stated otherwise, all media and buffer components were procured from Hi-Media. Laccase protein (powder form) isolated from *Trametes versicolor* (Specific activity ≥ 0.5 Umg^− 1^) was procured from Sigma-Aldrich.

### Immobilization protocol

#### Beads preparation

Using the cold oil spherification method, beads of varying concentrations (1.5, 2.0, 2.5, 3.0, and 3.5%) were prepared by dissolving known weights of agarose (Agarose M, Biogene, USA) in distilled water and boiled. At around 60^0^C liquid temperature, uniform droplets of the hot agarose were dispensed into an ice-cold mixture of filtered peanut (4 parts) and sunflower oil (1 part) using a needle-free syringe, to obtain around 3–3.5 mm agarose beads. The beads were then thoroughly given 4–6 washings with distilled water to remove any bound oil on the bead surface and stored in 0.4 M sodium acetate buffer (pH 5.2) until further use.

#### Beads functionalization

All the preliminary treatment steps for immobilization were performed both at 4^0^C and 28 ± 2^0^C. The agarose beads (all concentrations) were initially hydrated (2 g beads/10 mL buffer) with 0.4 M sodium acetate buffer (pH 5.2) for 2 h (80 rpm). Beads were then filtered and recovered for the next step. Functionalization of the beads was performed by adding the hydrated beads to 10 mL of 0.1 M sodium bicarbonate buffer (pH 10), along with 5 mL of 1 M ethylene diamine solution, and incubated for 3 h at both temperatures. This was followed by five washings with 0.4 M sodium acetate buffer (pH 5.2).

#### Beads activation

Functionalized beads were subjected to activation, by adding 1% glutaraldehyde in 10 mL of 100 mM HEPES buffer (pH 7.5) and incubated for 21 h (80 rpm) before washing with 0.4 M sodium acetate buffer (pH 5.2), 4–5 times to remove excess glutaraldehyde. The glutaraldehyde stock (25% v/v) was diluted using molecular-grade acetone (v/v) before activation.

#### Laccase protein cross-linking

Activated agarose beads (AAB) were treated with varying laccase concentrations containing 1–5 mg protein mL^− 1^ in 100 mM HEPES buffer (pH 7.5) and incubated for 2 days (80 rpm). 50 µL of the buffer was aspirated at regular intervals during the incubation period and activity measurements were made. One unit of laccase activity was defined as µmoles of ABTS oxidized min^− 1^. Incubation was stopped when no further laccase activity was recorded in the buffer.

#### Reduction of cross-linked beads

A reduction step was performed, at the end of the 32 h incubation, by adding 10 mg of sodium borohydride to the reaction mixture, and the solution with beads was further incubated for 0.3 h. The immobilized enzyme beads were then separated by filtration through a 120-mesh sieve size (0.125 mm), followed by five times washing with 0.4 M sodium acetate buffer (pH 5.2) and finally stored in 0.4 M sodium acetate buffer (pH 5.2) at 4^0^C.

### Protein assay

Protein concentration was determined according to the Bradford method^30^ using bovine serum albumin (BSA) as the standard.

### Adsorption experiments

Sorption isotherm models viz., Langmuir, Freundlich, and Temkin were used to find the relationship between the adsorbed laccase enzyme and the AAB. Different concentrations of laccase protein ranging from 1 mg to 5 mg mL^− 1^, immobilized on 3% AAB were considered for adsorption isotherm studies both at 4^0^C and 28 ± 2^0^C. To establish the model parameters, the experimental data were fitted to the above models using the OriginPro-nonlinear curve fitting application^31^.

The amount of adsorbed laccase, *qt* (mg/mg) was calculated using the following equation.

$$q_{t} = {\raise0.7ex\hbox{${(C_{o} - C_{t} )V}$} \!\mathord{\left/ {\vphantom {{(C_{o} - C_{t} )V} m}}\right.\kern-\nulldelimiterspace} \!\lower0.7ex\hbox{$m$}}$$

Where *q*_*t*_ is the amount of adsorbed laccase per unit weight of AAB at time t (mg/mg), *C*_*0*_ is the initial laccase concentrations (mg/mL) and *C*_*t*_ is the laccase concentration in the solution at time *t* (mg/ml), V the volume of the laccase solution (mL) and *m* is the mass (mg) of the agarose beads used. The activity of the immobilized laccase was calculated as U/mg of the immobilized catalyst.

### Efficiency, yield, stability

#### Recycling stability

The reuse potential of the immobilized laccase beads (IB) was determined by measuring laccase activity for up to 15 cycles using 2,2’-azino-bis-3-ethylbenzothiazoline-6-sulfonic acid (ABTS) as the substrate. After each successive cycle, IB was washed with 0.4 M sodium acetate buffer (pH 5.2), before assaying again. IB was stored at 4^0^C in 0.4 M sodium acetate buffer (pH 5.2), until further use. Laccase activity was determined by the oxidation of ABTS at 28 ± 2 °C^32^ by measuring the increase in absorbance (ε_420_ = 36000 M^− 1^ cm^− 1^) at room temperature after 5 min incubation. The reaction mixture contained 50 µL of 5 mM ABTS, 200 µL of 0.4 M Sodium Acetate Buffer (pH 5.2), and 0.1 g of IB, 0.1 g of empty beads without immobilization served as control. Enzyme activity was expressed in µmoles min^− 1^ mL^− 1^. One unit of laccase activity was defined as µmoles of ABTS oxidized min^− 1^.

#### Storage stability

The stability of the laccase-IB during storage at 4^0^C was monitored by measuring the activity loss due to deactivation at regular intervals. Percent stability was calculated using the following formula:

$$\:Storage\:stbility\:\left(\%\right)=\frac{Laccase\:activity\:\left({n}^{th}\:cycle\right)}{100}X\:Laccase\:activity\:\left({1}^{st}\:cycle\right)$$

#### Immobilization yield and immobilization efficiency

The percentage of total immobilized laccase activity from the free laccase solution was calculated using the following formula for immobilization yield.

$$\:Yield\:\left(\%\right)=100\:X\:\frac{Immobilized\:activity}{Initial\:activity}$$

The percentage of the total laccase bound to the AAB gave immobilization efficiency. The activity was calculated as U/mg of the immobilized laccase.

$$\:Efficiency\:\left(\%\right)=\frac{{C}_{e}}{{C}_{i}}X\:100$$

Where C_e_ is the concentration of laccase after immobilization while C_i_ is the concentration before immobilization.

### Treatment of crop residues with immobilized beads

Finger millet (FM) and paddy straw (PS) samples (10 g each), chopped to 2 cm, were first hydrated with acetone: water in a ratio of 1:1 for about 20 min. To this hydrated straw, 2 g of IB was added (2 g beads/10 g straw) and mixed at regular intervals through the 24 h incubation period. At the end of incubation, excess water was drained out and the beads were recovered for further use in the next treatment cycle. The above experiment in total was carried out in the working prototype of the batch enzymatic reactor detailed in Sect. Batch enzymatic reactor: working prototype. The treated straw samples were then finely powdered after drying at a constant temperature of 50^0^C in a hot air oven and characterized.

### Characterization studies

The powdered straw samples (FM and PS) along with their respective untreated controls and the agarose beads (free and immobilized) were characterized separately by FT-IR, SEM, and EDX analysis after freeze drying (using Christ Alpha 1–2 LD Plus lyophilizer) to evaluate the presence of laccase enzyme on the activated bead surface, as well as to assess the delignification of the crop residues after treatment. Instruments Perkin Elmer/Frontier for FT-IR analysis and ESEM- Quanta 200 (FEI Thermo Fisher) were used to perform SEM and EDX analysis of the free and IB. Samples were prepared by primarily mounting the freeze-dried beads on aluminium stubs using double-sided carbon tape, sputter coated with gold for high conductivity, and staged in the instrument chamber under a high vacuum to capture SEM images.

### Batch enzymatic reactor: working prototype

The straw samples using IB were treated in a working prototype of an enzymatic reactor, designed and developed using polypropylene plastic with a considerably low carbon footprint. The prototype is a miniature model confined to treating 10 g of the straw at a time, comprising four detachable parts: The valve unit, the separating unit, the collection unit, and the control unit. The valve unit includes a rotating valve that opens and closes the flow of liquid and solid particles. One end of the valve unit is connected to a wide chamber with a lid that unlocks to the top. Housed inside this is the impeller to rotate and mix the contents. The other end is connected to the second wide open chamber at the bottom, which sits on a 6.5 mm sieve tray, the separating unit. The collection unit at the bottom accommodates a 2 mm sieve tray, and a tap to drain the buffer out of the reactor. The control unit consists of a microcontroller and a motor. The code is fed to the microcontroller through an IDE (Integrated development environment). The microcontroller uses an Arduino UNO R3 microprocessor (Arduino with L298N Dual H Bridge DC/Stepper Motor Driver Controller Module) that aids in adjusting the motor rpm and reversing the direction of rotation of the impeller both clockwise and anticlockwise, every 10 s. A gear assembly is attached to the motor, with the speed counted through rotations min^− 1^ (RPM). The motor capacity is 6400 rpm at full power. The motor speed is varied using pulse width modulation (PWM), with a range from 0 to 255, currently calibrated to 100. The DC-geared motor is powered through a DC power supply. The unit also consists of a DC water pump, to fill and flush the system with water. Press-fit connectors are used for leak-proof water piping around the lid. Three controls: C1 through C3 correspond to power supply, motor, and water pump connectors respectively. The treatment of straw samples is carried out in the upper chamber of the valve unit, called the agitator consisting of an impeller that is mechanically driven via a micro-controlled electric motor. The straw and the IB are continuously mixed with the help of an impeller for 24 h to facilitate maximum enzymatic interactions with the substrate. At the end of the treatment step, the straw thus recovered from the reactor was mixed with common salt (1 g/kg straw)^33^, sun-dried for 24 h, and used for invitro digestibility studies.

### Estimation of lignin and dry matter digestibility

In vitro-dry matter digestibility (IVDMD) studies were carried out using rumen liquor collected from cannulated cows. A prior request form is submitted to the Animal facility well in advance for collection of the rumen contents and the experiment is planned in alignment with the availability of the same. Rumen liquor is collected, before feeding the cows, early in the morning into a CO_2_ flushed pre-warmed (37^0^C) thermos flask to mimic rumen conditions, brought to the laboratory, and strained through cheese cloth. Further, the strained liquor was processed immediately for use as inoculum in IVDMD studies. Acid detergent lignin (ADL) and IVDMD were evaluated as per the procedure detailed in Kumar et al.^26^.

### Data analysis

FT-IR spectral data was analyzed using OriginPro^31^ and Wiley’s KnowItAll ID expert software platform from Wiley (trial version). Peaks were identified in the lignin fingerprint region for the FT-IR spectrum between 1800 –800 nm using the quick peak gadget tool, with the baseline mode set to none (y = 0). The full-width half maximum (FWHM) values for the peaks were calculated using the peak analyzer for Gaussian fit. Hypothesis testing was performed using the one-sample t-test for FWHM values across peaks to check if there were any statistically significant differences between the FWHM values of the control and the treated straws. The FWHM values considered for each sample is an average of three replicates. One-way ANOVA was performed to evaluate the differences in control and treated paddy and finger millet straws for acid detergent lignin (ADL) and in vitro dry matter digestibility (IVDMD). These experiments were performed in five replicates.

### Ethical statement

This article does not contain any studies with human participants or animals performed by any of the authors. Rumen liquor (small volumes) was collected as part of the routine collection by the designated technical officer (veterinarian), to conduct in vitro dry matter digestibility experiments adhering to the guidelines and regulations as approved by the Institutional Animal Ethics Committee (IAEC). As no live animals were used in the study, an ethical review adhering to ARRIVE guidelines was therefore not required. No animal discomfort was caused by sample collection for the study.

## Results

**Fig. 1:**
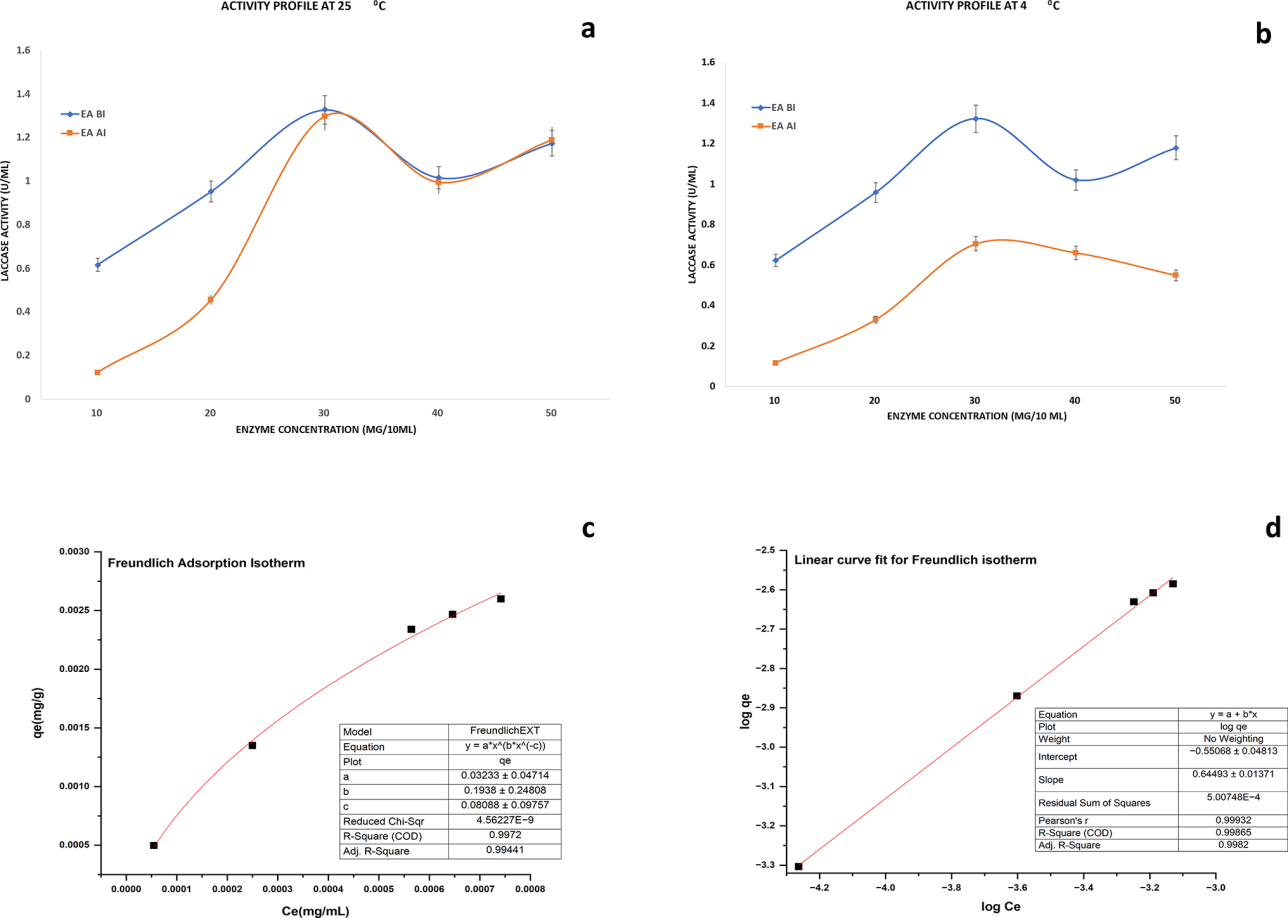
(**a** & **b**) Activity profile measurement: The enzyme activity for laccase enzyme (U/mL) before and after immobilization, for different concentrations of the laccase, adsorbed onto 3% agarose beads at **a**) 25 °C (**b**) 4 °C was monitored; (**c** & **d**) Adsorption isotherm model: Freundlich adsorption isotherm plot for the immobilization of laccase enzyme on activated agarose beads was generated using. OriginPro Nonlinear curve fitting application (Version 2023b). Graph ‘c’ represents a Non-linear curve plot while ‘d’ represents a Linear curve plot.

**Fig. 2:**
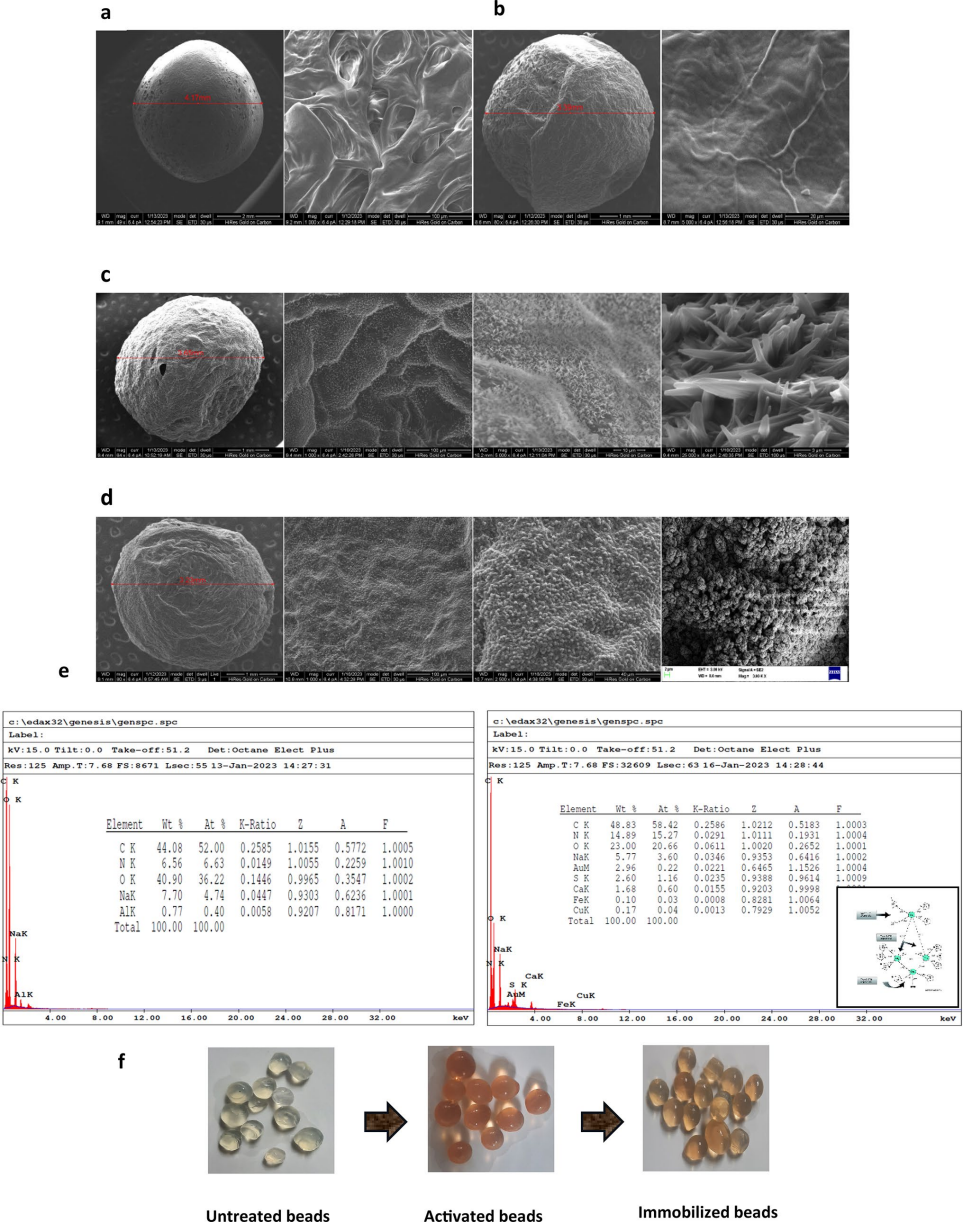
**Scanning electron micrograph studies of empty and laccase immobilized agarose beads **(**a**) 3% agarose bead (**b**) activated 3% agarose beads (**c**) Laccase immobilized on activated agarose beads at 4^0^C (**d**) Laccase immobilized on activated agarose beads at 25^0^C (**e**) EDX images of empty and laccase immobilized agarose beads with a mean diameter of 3.42 mm.{(ESEM- Quanta 200}(**f**) Immobilization of laccase enzyme on agarose beads. Images captured through the immobilization process across various stages.

The polysaccharide agarose is an excellent matrix for enzyme immobilization as it produces highly porous, mechanically resistant, chemically inert beads^34^. Different concentrations of agarose beads were prepared using the cold oil spherification method where the contact point of hot agarose and the cold oil transformed agarose into spherical beads. The strength of the beads (~ 35–40 mg per bead weight) improved gradually with the increase in the concentration of agarose polymer (Fig. 1). To adept them for the efficient immobilization of the laccase protein, the surface of the beads was chemically modified. The immobilization process was performed both at 4^0^C and 28 ± 2^0^C to establish an ideal temperature for effective immobilization. The entire process of bead modification at every step showed a change in the color of the treated beads. During the functionalization of the beads using ethylene diamine, the initially translucent beads turned ivory white after attachment of the amino groups. Next, pre-activation of the beads was performed using bi-functional reagent glutaraldehyde which turned the color of the beads to brown. The laccase protein when covalently cross-linked to AAB, modified the color of the beads to bisque on the immobilization of the protein (Fig. 2f).

### Characterization of adsorption isotherm

Effective immobilization is influenced by temperature and hence immobilization experiment was conducted both at 4^0^C and 28 ± 2^0^C to establish optimum conditions with maximum catalytic activity and binding capacity. Effective immobilization also depends on the adsorbent-to-adsorbate ratio. Laccase activity measurements before and after immobilization for different concentrations of the enzyme bound onto 3% agarose beads showed maximum adsorption at 28 ± 2^0^C for 3 mgmL^− 1^ enzyme concentration (Fig. 2a) showing adsorption experiment at room temperature to be more effective than at 4^0^C (Fig. 2b). The relationship between the amount of laccase enzyme adsorbed and the AAB was studied using three well-established physisorption isotherm models: the Langmuir, the Freundlich, and the Temkin. The data obtained through the immobilization experiment was used to fit all three models and the relationship of best fit between the laccase enzyme (adsorbate) in the buffer and the amount of enzyme adsorbed on the surface of the AAB (adsorbent) at equilibrium at a given temperature was characterized. The equilibrium concentration of the enzyme solution was considered after immobilization. Among the three isotherm models studied, the Freundlich adsorption model gave the best fit (R^2^ = 0.9987) (Fig. 2c,d) compared to the Langmuir adsorption isotherm model (R^2^ = 0.9711) (Fig S1a) and the Temkin isotherm model (R^2^ = 0.9579) (Fig S1b) (Supplementary data). The immobilization of laccase protein was directed towards the Freundlich isotherm model as the regression coefficient obtained was highest for this, predicting that the adsorption of laccase on AAB was a multilayer adsorption. Lavenberg Marquardt extended Freundlich adsorption isotherm equation y = ax^bx−c^ was used for iterations and the fit converged when the Chi-square tolerance value of IE-9 was reached. The Freundlich constant and n values were extrapolated from the intercept (-0.55068) and slope values (0.6449) respectively. The K_f_ value of 0.5766 measured adsorption capacity, with a 1/n value of 0.6449, determining the strength of the adsorbent material. The n value of 1.5506, indicated favourable adsorption.

### Characterization of the immobilized beads

Scanning electron micrograph (SEM) images were captured to observe the structural changes between the agarose beads before and after immobilization. Figure 1a-d displayed visible differences in the micrograph images where the outer surface of the empty beads appeared porous with a smooth surface; while in the AAB the surface was still smooth with the pores sealed. Irregular aggregates on the bead surfaces were observed after laccase immobilization due to enzyme coupling onto the activated beads. Differences were observed in the orientation of the coupled laccase proteins due to changes in the temperatures adapted for immobilization. Spikes were observed for covalently linked laccase at 4^0^C whereas rosette globular structures were visualized at room temperature. To support the observations made, the beads were also analyzed using energy dispersive x-ray spectroscopy (EDX spectra) to confirm the variations in the structural composition. During the immobilization process, laccases can have copper, zinc, and iron atoms, cross-linked to the agarose beads instead of necessarily having the classical four copper atoms in their active center. The presence of Cu, Fe, and S in the EDX spectrum (Fig. 1e) proved that the laccase molecule was bound to the AAB since neither the materials used in bead formation nor the solutions used in immobilization experiments contained Cu, Fe, and S elements. The appearance of Cu centers in the EDX data confirmed the effectual cross-linking of the laccase protein with glutaraldehyde retaining the active center of the molecules, thereby retaining its activity.

**Fig. 3:**
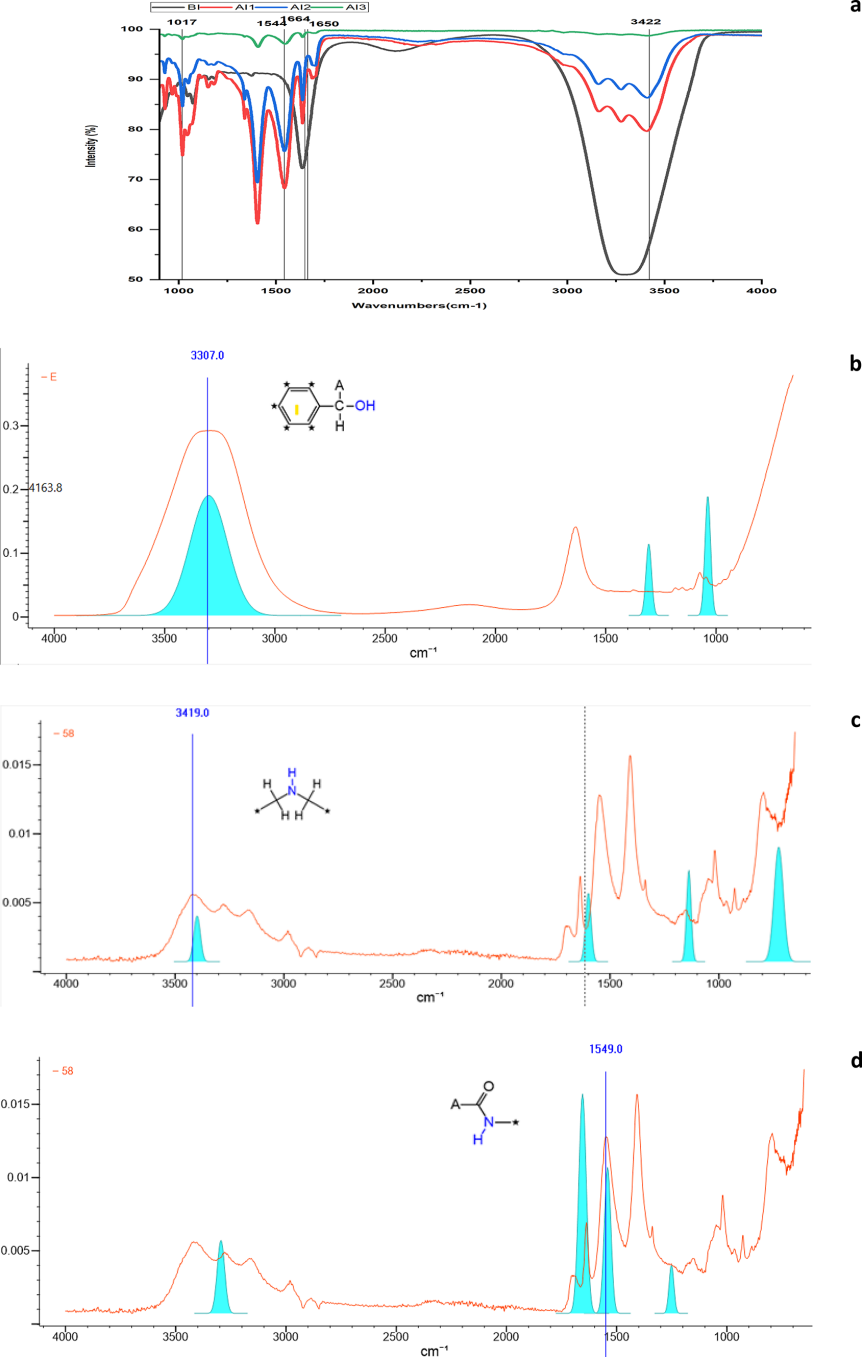
(**a**) Stacked FT-IR spectra of agarose beads, activated agarose beads before and after laccase immobilization to investigate the functional groups. Wiley’s KnowitAll analytical software was used to analyze and identify functional groups of the FT-IR spectrum (**b**) spectrum of empty activated agarose bead (**c** & **d**) spectrum of laccase immobilized activated agarose bead.

A Fourier transformation infrared (FT-IR) spectroscopy was performed to investigate the functional groups associated with the adsorbate and the adsorbent. Figure 3a to d represents stacked FT-IR spectra of the AAB before immobilization (BI) and after laccase immobilization (AI). Strong intensity at 3422 cm^-1^ indicated vibrations in the NH-OH regions while strong vibrational intensities at 1664 cm^-1^ represented CO-NH peptide linkage in the AI spectra. The presence of Amide I and Amide II is characteristic of the laccase protein, and the amide I and amide II bands at 1549 and 3419 cm^-1^ respectively confirmed its presence. Peaking at 3419 cm^-1^ was due to the C-N stretch of the amine vibration. The amide I band of the protein is the most sensitive region of the spectrum peaking at 1549 cm-^1^, which contributed to the C = O stretch vibrations in the peptide linkages. Amide I band is related to the secondary structure of the protein whereas Amide II is due to the in-plane NH bending and CN stretching vibrations. A sharp and strong peak was observed at 1017 cm^-1^ owing to the C-O-C linkage. The observed broadband in the spectrum at 3307 cm^-1^ of un-immobilized agarose bead is due to the OH groups present in the adsorbent.

### Evaluation of the immobilized beads

Laccase enzyme is capable of oxidizing various substrates using only oxygen as the co-substrate. The oxidation potential of the IB was evaluated using ABTS as the substrate. A bed of immobilized laccase agarose beads (30 g) was prepared using a separating funnel and 200 mL of 0.4 M Sodium Acetate Buffer (pH 5.2) was added. To this 1 µL of ABTS (5mM) was added to every 20 µL buffer. The liquid medium turned green from the colorless liquid on ABTS oxidation. At the end of each reaction cycle, the oxidized substrate was decanted, rinsed with distilled water and a second round of buffer-ABTS solution was added. The beads maintained their ABTS conversion potential even after 15 successive cycles of reaction. The same batch of IB was used for all 15 reactions. The specific activity of the enzyme recorded in the reaction medium was almost constant in all the reaction cycles (23U/mg), confirming stable covalent bond formation during the immobilization process.

### Immobilization yield and immobilization efficiency

**Fig. 4:**
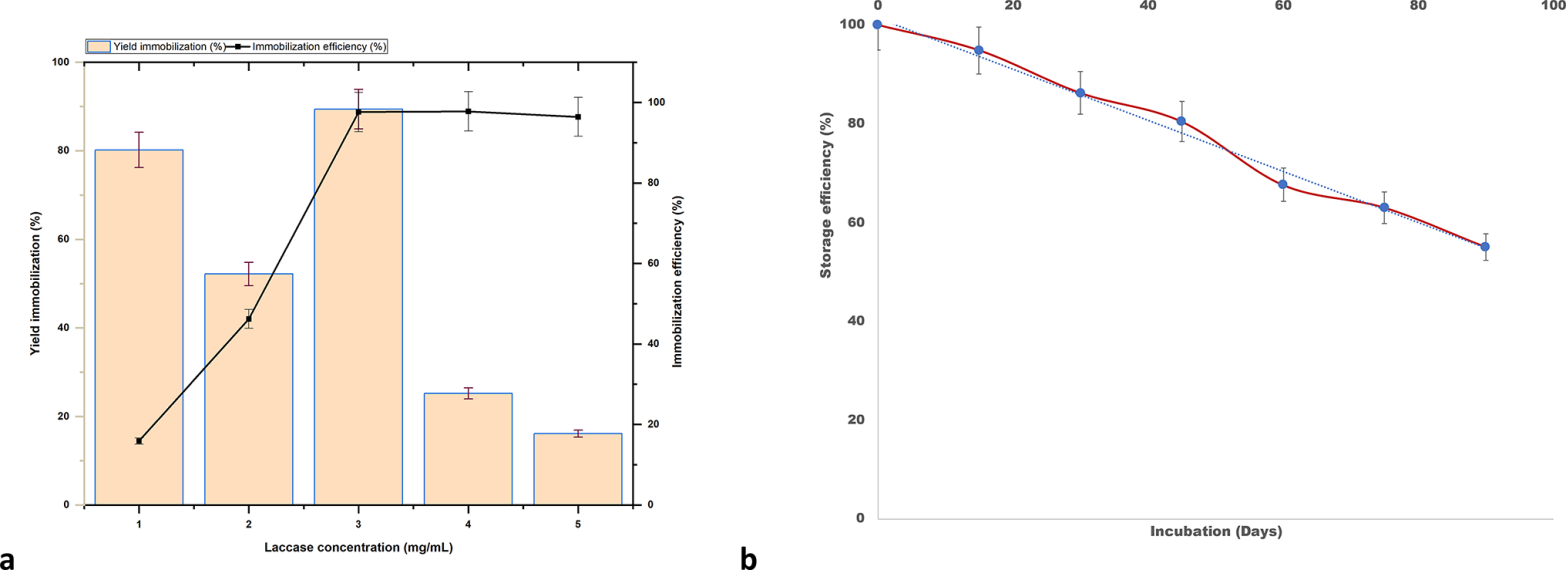
(**a**) Plot showing immobilization efficiency and immobilization yield (%) for different concentrations of laccase enzyme (mg/mL) immobilized on 3% activated agarose beads (**b**) Storage stability of immobilized laccase on activated agarose beads stored at 4^0^C. Residual enzyme activities were recorded periodically after storage in 0.4 M sodium acetate buffer (pH 5.2). The trendline in the graph shows decrease in laccase activity at a steady state over time. One unit of laccase activity was defined as µmoles of ABTS oxidized per minute.

Room temperature (28 ± 2^0^C) was ideally favorable for enzyme immobilization, as the beads were actively oxidizing the substrate ABTS, as gauged by the change in color of the medium qualitatively and measuring the enzyme activity quantitatively. The percentage yield and efficiency of immobilization are represented in Fig. 4a. Among the various concentrations of the laccase enzyme used for immobilization of AAB (3% w/v), 3 mg/mL enzyme concentration gave the best results, both in terms of immobilization yield and immobilization efficiency. The immobilization yield obtained was 89% with the immobilization efficiency of 97% suggesting successful immobilization of laccase on AAB with high catalytic activity.

### Effect of storage stability

To monitor the losses due to the deactivation of the laccase enzyme adsorbed onto an AAB support matrix, the stability of the enzyme was monitored for 3 months. Figure 4b depicts the storage stability of immobilized laccase agarose beads, retaining up to 94.87% of their initial laccase activity after 15 days and about 80.43% after 45 days of storage at 4^0^C. The IB retained 63.02% activity after 75 days of storage, demonstrating lower loss due to deactivation during the process of storage of IB at 4^0^C.

### Depolymerization of the crop residues

One of the major limitations in the use of biocatalysts is the challenges they pose in terms of separation of the enzyme, elevating the operation costs. The use of immobilized enzymes to a greater extent circumvents this problem by making the separation process feasible thereby increasing the reuse potential of the enzyme. Application of immobilized ligninolytic enzymes for depolymerizing lignin present in crop residues, especially for use in ruminant feeding has not been explored enough as (a) treating the bulky crop residues is challenging (b) recovery of the beads is difficult (c) beads in case consumed accidentally along with the treated straw, should not be toxic to the animal. Considering the aforementioned challenges and to come up with a sustainable safe green technology to treat crop residues, a working prototype of a simple batch enzymatic reactor was designed.

**Fig. 5:**
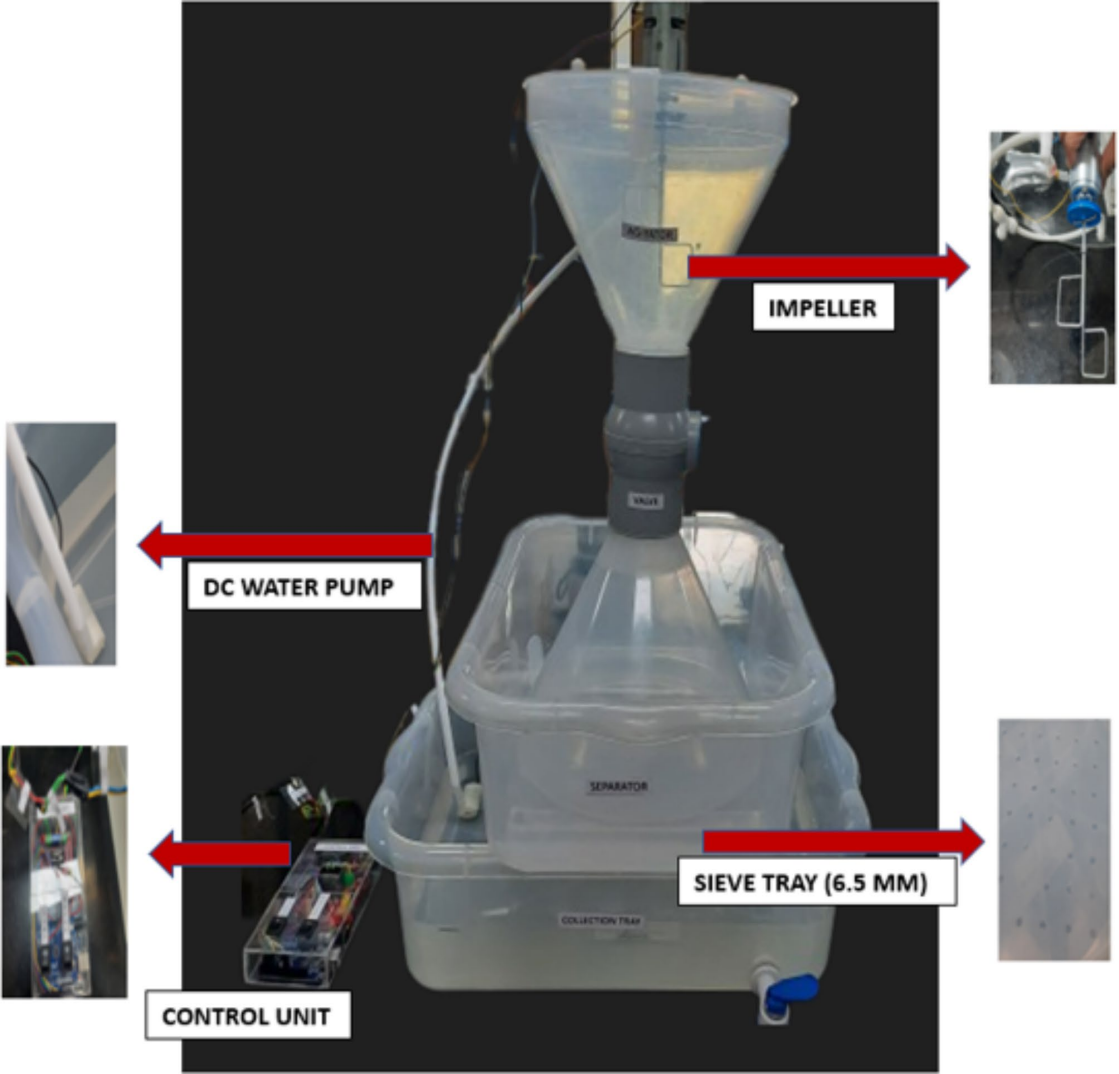
Enzymatic reactor prototype designed and developed for the treatment of crop residues using laccase immobilized agarose beads.

Recalcitrant lignin in the crop residues needs continuous contact with the catalyzing enzyme for efficient delignification to take place. The prototype was designed for laccase IB to continuously oxidize the straw samples with controlled operations, ensuring minimum pressure on the beads to avoid rupture (Fig. 5). The enzymatic reaction was carried out in the upper chamber of the valve unit, called the agitator where 2 cm length straw samples (10 g), buffer, and enzyme beads were added to initiate the oxidation/reduction reactions. Mixing in the agitator of the enzymatic reactor is provided by an impeller mechanically driven via a micro-controlled electric motor. After 24 h of the reaction cycle; on opening the chamber valve; buffer, beads, and straw pass through the lower chamber of the valve unit. Straw is retained on the 6.5 mm sieve tray, while beads pass through it further down, along with buffer. Beads are separated in the second sieve tray (2 mm) and the spent liquid is collected at the bottom in the collection tray, and by using the tap provided, is emptied from the reactor.

### Characterization of crop residues

#### SEM/EDX analysis

**Fig. 6:**
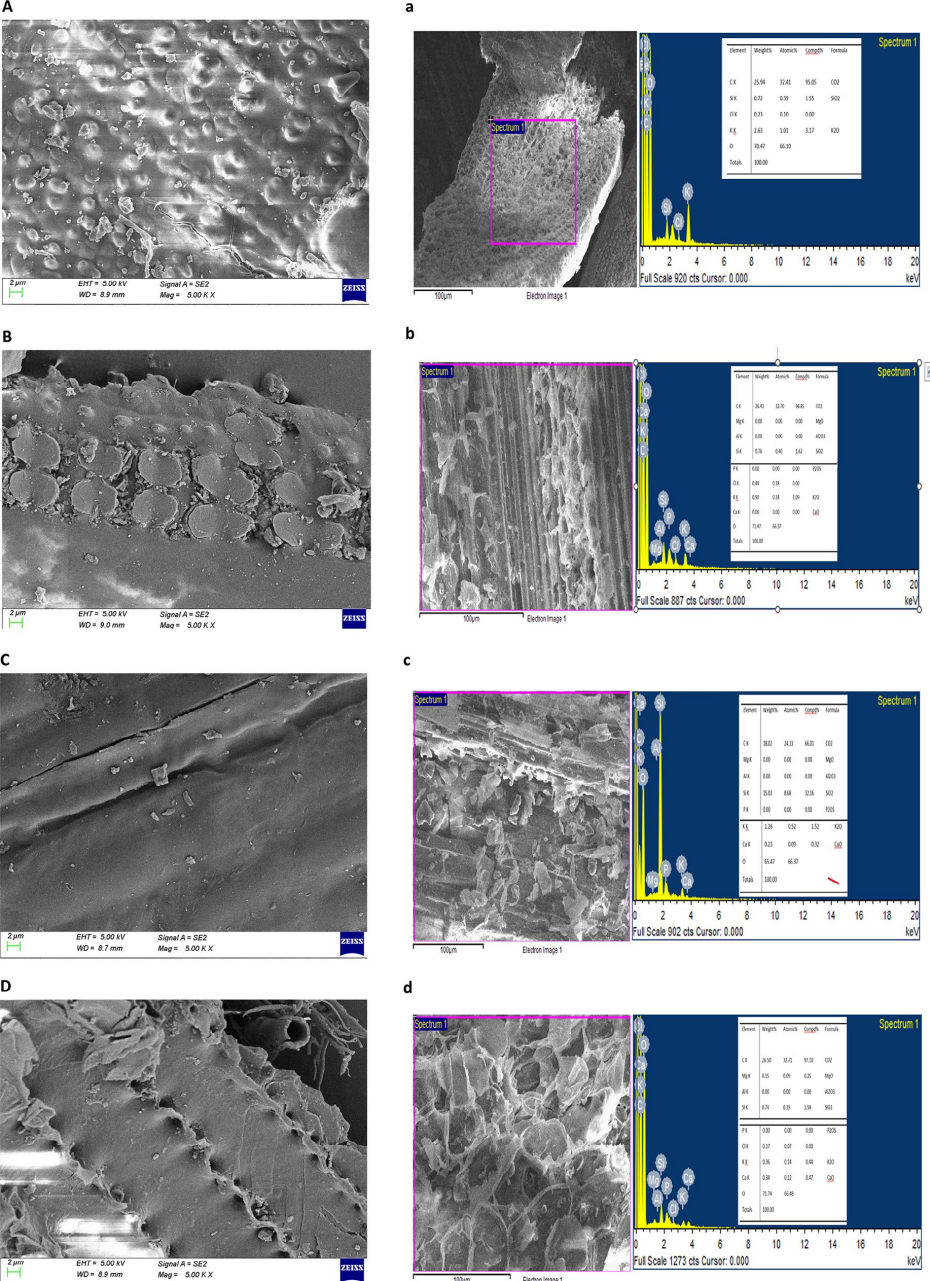
**SEM and EDX images of crop residues with and without treatment** (**A**), (**B**), (**C**), (**D**) represents SEM images of untreated and treated straws at 2 μm resolution **a**, **b**, **c**, **d**) represents EDX spectra of untreated and treated crop residues captured at 100 μm resolution A & (**a**) untreated paddy straw B & (**b**) paddy straw treated with immobilized laccase beads C & (**c**) untreated finger millet straw D & (**d**) finger-millet straw treated with immobilized laccase beads. (Ultra 55 FESEM (CarlZeiss)).

Elemental analysis of untreated (control straws) and laccase immobilized AAB-treated PS and FM straws (treated straws) was carried out using EDX-XRF. The relative concentrations of Mg, Al, Si, P, Cl, K, Ca, O, and C were compared. SEM and EDX images of control and treated straw samples are depicted in Fig. 6. The oxygen concentration for pretreated straws of FM and PS control was 65.47% and 70.47% respectively while the concentrations increased to 71.74% and 71.47% respectively for the straws treated with IB. Likewise, ‘C’ concentrations also increased to 26.50 and 26.43% in immobilized laccase-treated FM and PSs respectively from control straws with 18.02% in FM and 25.94% in PS. These results indicated a corresponding increase in the carbohydrate content of the crop residues of both FM and PS after treatment using IB. The weight percentages of Si, Cl, and K in untreated PS were 0.72, 0.23, and 2.63% respectively changing the concentrations after treatment to 0.76, 0.44, and 0.90%. The concentrations of Si, K, and Ca in treated FM straw were 0.74, 0.36, and 0.34 respectively, while Si, K, and Ca weights were 15.03, 1.26, and 0.23% in control straw. No visible weight of Cl was recorded in the control while the treated FM straw showed 0.17% Cl. Both the straws showed a decrease in potassium concentrations after treatment while an increase was observed in Cl concentrations after treatment. An increase in weight percentages of Ca and Cl from untreated to treated straws indicated the release of bound elements after enzymatic treatment.

#### FT-IR analysis

**Fig. 7:**
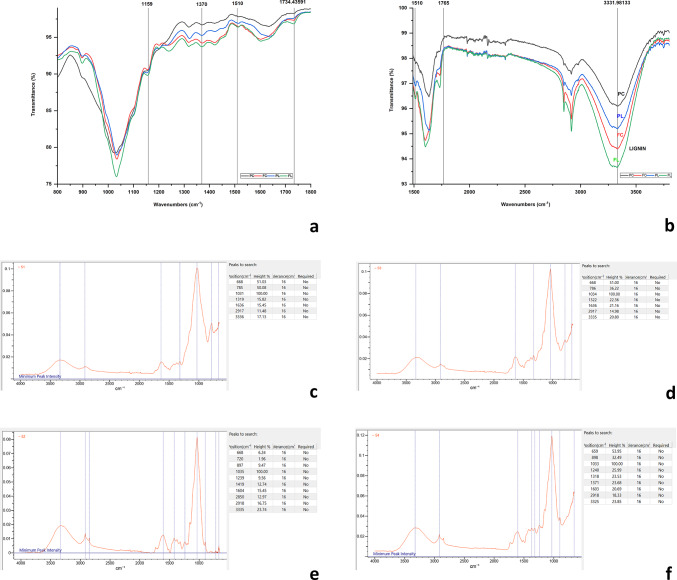
FT-IR spectra of untreated and immobilized laccase-treated paddy and finger millet crop residues (**a**) 800–1800 cm^− 1^ wavenumber range (**b**) 1500–4000 cm^− 1^ wavenumber range (**c**-**f**) Area of peaks calculated using Wiley’s KnowitAll analytical software (**c**) Paddy control (**d**) Paddy straw treated with laccase immobilized agarose beads (**e**) Finger millet control (**f**) Finger millet straw treated with laccase immobilized agarose beads.

FT-IR spectra of untreated and immobilized laccase-treated PS and FM crop residues are represented in Fig. 7. The IR spectra of PS showed strong O-H stretching and -C-H stretching absorptions at 3336 cm^-1^ and 2917 cm^-1^ respectively. These two strong absorptions are because the three major components in biomass (cellulose, hemicellulose, and lignin) with hydroxyl groups and many C-H bonds in their structures. The absorption at 1636 cm^−1^ and 1765 cm^-1^ represented asymmetric stretching bands of the carbonyl group in hemicellulose and lignin. The CH_2_ wagging in cellulose and hemicellulose and the C-O stretching of the substituted aromatic units such as syringyl and condensed guaiacyl units were observed at 1319 cm^−1^ for control and at 1322 cm^-1^ for laccase-treated straw. The intensity of alkali lignin at 1636 cm^−1^ increased after laccase treatment indicating the formation of small molecule lignin. Contrary to this, the IR spectra of FM straw showed strong C-H stretching absorbance at 2918 cm^−1^. The adsorption peak at 1240 cm^−1^ for laccase treated straw (1239 cm^-1^ for the control), showed an increase in intensity due to the stretching vibrations of the hemicellulose acetyl esters, which significantly changed after laccase treatment. The band significantly changed at 1734 cm^-1^, which is mainly attributed to the C=O vibration in acetyl and p-coumaryl groups in lignin, indicating that ester bonds were cleaved in lignin. There is a shift in peak position from 668 cm^−1^ in control to 659 cm^-1^ in treated straw with the increase in intensity as observed from the height% of 6.24 to 53.95%. The bands at 897 cm^−1^ and 898 cm^−1^ respectively for control and laccase-treated samples arose from C-O-C stretching at the β (1–4), glycosidic linkages in cellulose and hemicelluloses. The increase in the intensity of the peaks at 898 cm^−1^ suggested that most of the polysaccharides in the lignocellulose were not degraded during laccase treatment.

The fingerprint region (1800 –800 cm^− 1^) of the FT-IR spectrum was analyzed to identify the structural changes in the intensities of spectral peaks unique to the lignin polymer distribution as lignin in the straws is not concentrated in one single region. To identify the total number of peaks in the fingerprint region of the FT-IR spectra corresponding to control and IB-treated FM and PS, peak analysis was performed using a quick peaks gadget by picking peaks in the region of interest (ROI). Peak analysis of this region gave 40 peaks for FM control (FM_C), while 33 in the treated straw (FM_T). Likewise, 61 peaks were present in PS control (PS_C), while 40 were identified in the treatment straw (PS_T). PS peaks (34%) and FM peaks (17,5%) disappeared in the treatment group respectively. Apart from the fingerprint region, the spectrum > 1800 cm^− 1^, up till 4000 cm^− 1^, there were 184 and 174 peaks in FM_C and FM_T, while 266 and 235 peaks in PS_C and PS_T respectively. This observation, clearly demonstrated that there are lignin monomers along the entire spectrum associated with mostly hemicellulose and cellulose, which contributed to the reduction in the total number of peaks. Also, there are continuous stretching, wagging, and bending vibrations observed along the length of the spectrum as observed with the shift in frequencies, in the treatment samples compared to the control for both the crop residues studied. Analysis of the FT-IR spectrum of the FM straw, showed peaks 740 cm^− 1^, 756 cm^− 1^, 1631 cm^− 1^, 1649 cm^− 1,^ and four peaks between 1710 and 1729 cm^− 1^ in the control while it disappeared in the laccase-treated straw. A similar trend was observed for PS as well. PS showed three peaks between 719 and 730 cm^− 1^, three peaks between 1561 and 1576 cm^− 1^, four peaks between 1619 and 1635 cm^− 1^, and eight peaks between 1720 and 1787 cm^− 1^ in the control straw, which was not seen in the treated sample. This clearly showed that lignin concentrated in these fingerprint regions was depolymerized and broken down into monomers. The density plot and the horizontal step graph (Fig S2) (Supplementary data) also validated the above observations.

**Fig. 8:**
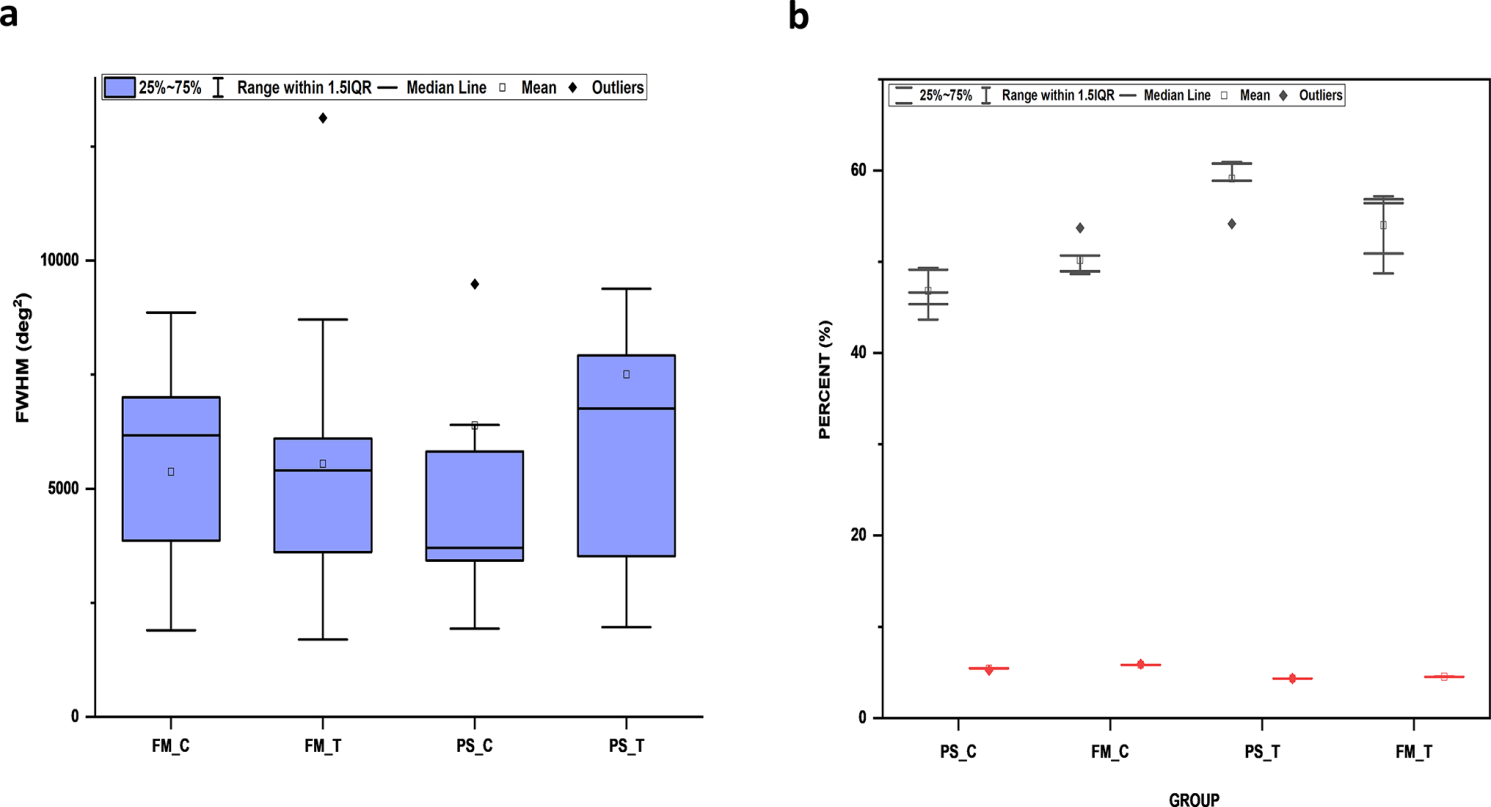
Comparative study of the full-width half maximum (FWHM) values of peaks identified in the fingerprint region of the FT-IR spectrum of the immobilized laccase-treated crop residues (**a**) The boxplot represents FWHM values of straw samples for finger millet control (FM_C), finger millet immobilized laccase treated (FM_T), paddy control (PS_C), paddy immobilized laccase treated (FM_T) (**b**) The group column scatter categorical graph, shows distribution of acid detergent lignin (ADL) and in vitro dry matter digestibility (IVDMD) of paddy and finger millet straws, after treatment using immobilized laccase beads. Bars represent the standard error of means from five replicates.

To establish a statistically significant difference between the FWHM values of the control straw and the laccase-treated straw samples, a one-sample t-test was performed across FWHM values of FT-IR peaks for FM and PSs (Fig. 8a) at 0.05 confidence level. The t statistic of FWHM values for treated FM straw was 6.69348 (M = 5544.9; SEM = 828.4; df = 12) with *p* < 0.0001 while the t statistic was 8.38475 (*p* < 0.0001) for the FWHM values of the control FM straw (M = 5372.04; SEM = 640.6; df = 12) showing a significant difference in the population mean from the test mean at 95% CI. The same trend with increased t statistic of 4.10967 for the FWHM values obtained for FT-IR peaks in the treated PS (M = 7504.2; SEM = 1825.9; df = 12) with *p* = 0.001 was observed when compared to the FWHM peak values obtained for control PS with a t statistic of 2.87344 (M = 6384.5; SEM = 2221.8; df = 12) with *p* = 0.01, also showing a significant difference in the population mean from the test mean at 95% CI. One sample test for variance at 0.05 CI also showed significant variances in the population after treatment, with a chi-squared value of 1.07058E8 (*p* < 0.0001) for IB-treated FM straw and a chi-squared value of 5.20137E8 for IB treated PS (*p* < 0.0001) with 12 degrees of freedom. The variations of the chi-square values obtained for one sample variance test comparing the FWHM values of the FM straw and PS can be attributed to the variations in the lignin composition of both straws. These differences observed in the chemical and structural characteristics of lignin between the straws can be accredited to the genetic variety of the crop and the surrounding growth environments^35^ contributing to the differences in phenotypic expressions.

To evaluate the absorption of carbohydrate energy by the ruminants and to monitor the extent of lignin depolymerization, invitro dry matter digestibility and proximate composition analysis were conducted for both paddy and finger millet straws treated with laccase immobilized beads. Figure 8b shows the distribution of ADL and IVDMD in treated straws compared to the controls visually displaying the differences in population means between the groups, where there was a decrease in ADL percentage to 19.96 and 22.64 in treated paddy and finger millet straws respectively. Analysis of Variance showed a significant difference in the population means for both ADL (t-statistic = 13.92; *p* = 8.01 × 10^− 4^) and IVDMD (t-statistic = 21.19; *p* = 2.29 × 10^− 4^) in finger millet and paddy straws from the test mean at 95% confidence interval. There was an increase in digestibility by 26.21% in treated paddy straw and an increase of 7.62% in treated finger millet straws over the wet controls with no treatment, clearly showing the positive influence of using enzyme beads in salvaging the carbohydrate energy of the crop residues because of sufficient lignin depolymerization.

## Discussion

Sustainable enzymatic methods for lignin degradation could unlock the full potential of polysaccharide digestion in ruminants, addressing the issue of underutilized energy. Laccase, a lignin depolymerizing enzyme, oxidizes molecules containing an aromatic ring substituted with an electron-withdrawing group such as phenols, cresols, chlorophenols, aryl diamines, aromatic amines, and polymethoxybenzenes^36^ and are hydrophilic. Immobilization of oxidoreductases such as polyphenol oxidases and peroxidases on insoluble carriers facilitates the formation of stable systems of the electron chain^37^ unlike applications using soluble enzymes which challenge the stability^38^. In the present study, the laccase enzyme was immobilized onto AAB by covalent cross-linking, which forms stable and rigid structures under appropriate conditions. Macroporous agarose beads were prepared via cold oil spherification and functionalized with ethylene diamine. At pH 10.0 the glyoxyl groups on agarose react with amine groups, blocking the support reactive groups by the aminated compounds^39^. Glutaraldehyde was then used to cross-link laccase onto the bead surface. The addition of glutaraldehyde forms a spacer between the protein surface and the inert matrix creating enough room for enzyme substrate interactions. Both ends of glutaraldehyde react with amines, forming an imine or azomethine under ideal conditions, substituting the carbonyl group when glutaraldehyde comes in contact with the amine groups of the functionalized beads or the amine groups in proteins due to Schiff’s base formation as well as Michael’s adducts^40^. Glutaraldehyde mainly cross-links with thiols, phenols, and imidazoles of proteins apart from primary amino groups on lysine residues. Schiff bases are unstable under acidic conditions and also covalent attachment is initiated when a moderate ionic strength is used^41^. Sodium borohydride was added to stabilize the Schiff base linkages producing a secondary amine^42^. A yellowish-orange color change after immobilization indicated successful protein binding.

Enzyme immobilization improved the stability of the laccase enzyme forming strong covalent bonds. This eventually decreased the denaturation enthalpy by reducing the total number of hydrogen bonds^43^ between the functional groups of the enzyme and the support matrix^44^. In addition, the agarose percentage influenced higher geometric enzyme-support compatibility^45^. Stability was also influenced by the use of organic solvent acetone in preparing working concentrations of glutaraldehyde. Milstein et al.^46^ recorded great stability of laccase immobilized in Sepharose-CL-6B glass beads when organic solvents like hexane, tetrahydrofuran, and dioxane were used. Kashefi et al.^47^, introduced amino functional groups on graphene oxide (GO) nanosheets by reacting them with APTES in an ethanol solution. Successful immobilization was also achieved by the strength of the support carrier and enzyme loading that eventually established a stable bond between the two avoiding nonspecific interactions^48^ and creating an adsorbate-adsorbent equilibrium.

To determine the amount of laccase enzyme that binds to the adsorbent beads, adsorption equilibrium was studied. The Freundlich isotherm model, with constants K_f_ (0.5766) and n (1.5506), indicated that the enzyme adsorbed well to the beads. A higher K_f_ value means more enzyme was adsorbed, while a higher n value suggests a heterogeneous surface with varying binding strengths. Similar results were obtained by Boparai et al.^49^ when the adsorption of lead (II) ions from aqueous solutions using coin dust and its modified extract resins were investigated. Gilani et al.^50^ studied the immobilization of lipase on activated chitosan beads which also followed the Freundlich isothermal model. While the Langmuir model suggests a single layer of molecules adsorbing, the Temkin model proposes that the binding strength of enzyme molecules decreases as more layers form on the adsorbent. Attaching enzymes to different support materials can make them last longer and withstand higher temperatures. This is because immobilization helps stabilize the protein structure through strong covalent bonds. Laccase, covalently immobilized on polyimide aerogels modified by ethylenediamine showed only 22% of its initial activity after six cycles^51^. In the extant study, the storage stability of the laccase was recorded regularly for 90 days. Only a little more than 10% decrease in activity was documented after 30 days of incubation. The IB retained 63.02% activity even after 75 days of storage. Zdarta et al.^52^ spectroscopically studied the storage stability of immobilized bacterial laccases over 75 days using ABTS as substrate recording 70% retention in initial activity after 30 days of storage at 4 °C. The observations made establish the fact that cross-linking of laccase enzyme to the AAB has made it more resistant to denaturation improving stability effectively. To check for the enzyme recoverability and reuse potential, the immobilization yield and immobilization efficiency were evaluated. The experiment gave an immobilization yield and efficiency of 89% and 97% respectively. Oliveira et al.^53^ observed an immobilization efficiency of 86% with significant improvement in the thermal stability compared to the free protein when the commercial enzyme DepolTM was immobilized by multipoint covalent attachment onto agarose beads. Similarly, immobilization yield and immobilization efficiency of 73.2% and 78.4% respectively were obtained by using an aldehyde functionalized support matrix^54^. This clearly stated that the covalent bonds formed due to the cross-linking of the laccase enzyme with the activated beads are stable and cannot easily leach out from the matrix which allows the enzyme to be used repeatedly over a long time.

The beads were analyzed using spectroscopic techniques to understand their physical properties. SEM images showed a change in the appearance of the beads after the laccase enzyme was attached. The temperature at which the enzyme was attached affected the surface structure of the beads, with 4^0^C resulting in a different arrangement of the enzyme on the bead surface compared to 28 ± 2^0^C. Yusdy et al.^55^ observed enhanced longevity, operational stability, and reusability of the enzymes because of orientation shifts in the covalent binding of immobilized enzymes on magnetic nanoclusters. Thiyagarajan et al.^56^ observed the presence of Fe-O bond characteristic of Fe_3_O_4_ in vibrational peaks of the FT-IR spectrum, validating the adsorption of laccase. Zhang et al.^27^ also recorded characteristic functional groups from the FT-IR spectrum of Fe_3_O_4_@CS@laccase composite nanoparticles at 576 cm^− 1^, 571 cm^− 1^, and 580 cm^− 1^ confirming the presence of laccase. The FT-IR spectrophotometer used for analysis in the present study measured the light absorption in the scanning range of 4000–600 cm^-1^. Therefore, the spectral details at wavelengths < 600 cm-1 could not be captured. However, the existence of Cu and S elements in the EDX spectrum validated the presence of laccase protein which is otherwise not present in the AAB alone. The detection of amide I and amide II regions in the FT-IR spectra and the appearance of Cu in the elemental analysis of the EDX spectrum confirmed the efficient immobilization of laccase.

Using immobilized laccase enzymes (IB) to break down lignin in crop residues for ruminant feed hasn’t been explored much. The bulky nature of the crop residues makes enzymatic treatment difficult. To improve the process, the PS and FM straws (2 cm in length) were mixed with acetone water first before treatment with laccase IB. Water helps the enzymes reach the plant cell walls, while acetone hydrates the residues and reduces the formation of unwanted byproducts, leading to better lignin breakdown. Previous research has shown that using acetone can significantly improve enzymatic hydrolysis by up to 94.2%^57^. This is a promising method because both acetone and water can be easily recycled, making it environmentally friendly.

There is a need for a proper operational protocol to be in place for using enzyme beads to treat crop residues, separate the treated straw, and recover the beads for reuse. Hence, a simple working prototype of an enzymatic reactor that could be used in the field to ensure efficient mixing and distribution of the enzyme beads was designed. Paddy straw and finger millet straw were treated separately in batches using the enzyme beads. The straws and beads were mixed in the reactor for 24 h before the beads were recovered for reuse in another cycle. The same set of beads was used for four cycles, and 98.8% of the beads were easily recovered.

As the lignin moieties in the straw are widely distributed, different analytical methods were used to analyze the effect of enzyme treatment on the crop residues. Lignin extracted from crop residues is not commercially available and existing kraft lignin and other forms of processed lignin cannot be considered as a comparative model for crop residue assessment, as they are structurally and functionally different. Spectroscopic analyses such as SEM, EDX, and FT-IR were used to characterize the extent of delignification in the dried and powdered treated straws. The increase in C and O elements after laccase treatment in both straws is indicative of exposure to polysaccharide components due to delignification. High amounts of potassium and low amounts of sodium decrease the absorption of magnesium and calcium in the rumen^58^. Also, excessive levels of potassium pose a dietary risk for milk fever^59^. Therefore, the decreased potassium levels observed after straw treatment with IB are an additional advantage. Calcium chloride is used in animal feed as an anionic salt as well as a lactating dairy cattle feed supplement as an energy source to increase milk productivity^60^. Chlorine is added to ruminant diets to help with fat metabolism and improve nutrient digestion, feed efficiency, milk production, and milk quality. Ruminants are regularly fed on concentrates and forage to supplement the nutrients they need. The increase in C, O, Ca, and Cl along with the decrease in K levels, clearly illustrates the reduction in cost prices as this ensures proper absorption of minerals, limiting the dependability on feed supplement resources as more of these elements are bioavailable through laccase treatment from the straws.

FT-IR spectroscopy was used to examine the functional group associated with the molecular bond and interpret the change in the hybridization state/electronic distribution of the molecular bonds. This was determined by analyzing the intensity and position of the peaks in the spectrum. Lignin contains different functional groups, such as methoxyl, carbonyl, carboxyl, and hydroxyl, which are attached to aromatic or aliphatic parts of the molecule^61^. The amount and combination of these groups affect the composition and structure of lignin. The intensity of the peak at 1267 cm^− 1^ changed slightly after laccase pretreatment, which suggested that the carbonyl in guaiacyl ruptured during the pretreatment. The adsorption peak at 1240 cm^− 1^ was from stretching vibrations of the hemicellulose acetyl esters, which significantly changed after laccase and LMS treatment. The carbohydrate polymer cellulose prominently showed up near 1660, 1280, and 840 cm-1 with hydroxyl groups converted into nitrate ester groups to give cellulose nitrates (nitrocellulose). This suggested that most of the polysaccharides in the lignocellulose were not degraded during the laccase pretreatment process confirming the selective degradation of lignin by IB. The FT-IR spectral data that was obtained was further processed by detecting the peaks obtained in the signal and comparing peaks, confirming significant variations in the peak areas after crop residue treatment.

A rough estimate of the economic viability of the immobilized laccase enzyme used for the delignification of FM and PSs was extrapolated, from the knowledge of unit operations worked out for cellulases^62^ The cost of laccase enzyme poses an impediment to industrial-scale applications. However, immobilized laccases with reuse potential bring down the costs to a substantial level, paving the way for large-scale applications. The unit cost of 1 g of laccase is around $118 (Sigma-Aldrich). The cost of immobilization for 2 g of beads (~ 30 mg laccase) is $3.56. Per treatment cost is $2.51. Considering the reuse potential of IB, it can be estimated that the cost of $83.6 could be incurred to treat 12–15 kg of dry fodder which is the average consumption of a cow weighing ~ 500 kg. The simple design proposed by us brings down the reactor cost significantly. The treatment cost can be substantiated by the assumption that bulk purchases of supplies would make the purchases far cheaper. In addition, feeding the ruminant cattle with laccase-treated crop residues along with a concentrate mixture will increase and improve milk production gaining extra income to the farmer by $4.73 because of premium quality milk, along with weight gain getting a total profit of around $6.78. Based on the findings of the current study and extrapolated from the theoretical evidence obtained on studies with cellulases, field experiments further with lactating animals will conclusively prove the benefits achieved in feeding pretreated straw to the ruminants. In addition, from the environmental perspective, improved digestibility as envisioned from the IVDMD values obtained shall reduce methane emissions.

During the process of immobilization of beads, chemicals like glutaraldehyde and sodium borohydride were used in trace quantities. As a final processing step, before feeding the treated crop residues to animals, it’s recommended to mix them with salt and dry them in the sun. This helps to minimize the risk of animals consuming any remaining beads that may have escaped the recovery process. Adding salt to the feed can improve its taste, encouraging animals to eat more and chew their food thoroughly. Salt is often used in animal feed to provide essential sodium and chlorine. A dairy cow (~ 650 kg) producing around 31 kg of milk needs a sodium intake of 231 MJ/day to meet the energy demands^63^. In addition, the salt neutralizes sodium borohydride used to reduce carbonyls during the immobilization process. Sodium borohydride decomposes in neutral or acidic aqueous solutions and is stable at pH 14^64^. Also, sodium in the form of salts improves the appetite for foods by increasing palatability translating into weight gain, improved fertility, and increased milk production. Glutaraldehyde has an estimated half-life in an air of 16 h. Sun drying the straw for 24 h takes care of any traces of unmodified glutaraldehyde present. The National Toxicology Program (NTP) does not list glutaraldehyde on the known or suspected cancer-causing substances^65^ and suggests through animal studies that the route of exit of glutaraldehyde from the bloodstream is either through feces or urine or exhaled air.

Since most of the beads (98.8%) are recovered, only a few might remain on the treated straw. Feeding the dried straw should be safe and not pose any health risks. Very few reports are available in the literature on the use of immobilized enzymes in the feed and fodder sector. Lopes et al.^66^ used zeolite modified with iron for immobilization of phytase enzyme for hydrolysis of phytate in soya bean meal, wheat bran, etc. to make the phosphorus more bioavailable for monogastric animal productivity. The proposed processing method of pretreating crop residues using immobilized laccase enzyme in a simple reactor made the nutrients more accessible. This could lead to more efficient and cost-effective use of lignocellulosic materials. Additionally, using immobilized laccase beads to break down the lignin in crop residues improved the digestibility of paddy and finger millet straws, which could influence better ruminant productivity and performance.

## Conclusion

A cost-effective, scalable method for enzymatic delignification of crop residues using immobilized laccase was developed. The proposed reactor design, employing covalently cross-linked laccase on activated agarose beads, offers a sustainable and replicable approach for enhancing ruminant productivity. This research demonstrates the potential of immobilized enzymes for large-scale bioconversion of lignocellulosic materials, contributing to a greener and more efficient agricultural production system.

## Electronic supplementary material

Below is the link to the electronic supplementary material.

Supplementary Material 1

## Data Availability

The data that support the findings of the study: the firmware (the code flashed into the microcontroller) of the batch enzymatic reactor prototype, video clips of ABTS oxidation (V1), and crop residue treatment using the reactor (V2) are available at https://github.com/vidyaprad/Enzymatic_Reactor as Enzymatic_Reactor on GitHub. Further queries can be directed to Dr. Vidya Pradeep Kumar at vidyaprad@gmail.com.

## Abbreviations

AAB: Activated agarose beads
IB: Immobilized laccase beads
BI: Laccase beads before immobilization
AI: Laccase beads after immobilization
FM: Finger millet straw
PS: Paddy straw
SEM: Scanning electron micrograph
EDX: Energy dispersive x-ray spectroscopy
FT-IR: Fourier transformation infrared spectroscopy
NDF: Neutral detergent fiber
ADF: Acid detergent fiber
ADL: Acid detergent lignin
IVDMD: In vitro dry matter digestibility
