# Supplementary material for: Sustainable pretreatment method of lignocellulosic depolymerization for enhanced ruminant productivity using laccase protein immobilized agarose beads
Source: Sci Rep. 2024 Oct 27;14:25617. doi: 10.1038/s41598-024-76278-0 (PMC11514234; doi:10.1038/s41598-024-76278-0)
Supplement: Supplementary file 1 — Supplementary Material 1 [file 41598_2024_76278_MOESM1_ESM.docx]

Supplementary data

Legends for supplementary figures:

Fig S1: Adsorption isotherm models for immobilization of laccase enzyme on activated agarose beads a) Langmuir isotherm model b) Temkin isotherm model

### Fig S2: a: A **probability d**ensity distribution with rug plot created to study the influence of immobilized laccase treatment on lignin depolymerization of finger millet and paddy straws compared to untreated straws. The smoothness of the lines is controlled by a bandwidth using a non-parametric kernel density estimate, where a continuous curve (Gaussian bell curve) is drawn at every individual peak point and summed up to a single smooth density estimation.The X-axis represents the wavenumbers associated with the peaks of the FT-IR spectrum in the fingerprint region (1800-800 cm^-1^) in the finger millet control (FM_C), immobilized laccase treated finger millet straw (FM_T), paddy control (PS_C), immobilized laccase treated paddy straw (PS_T). The Y-axis represents the probability density (probability per unit on the x-axis) function for the peak density estimation. The rug shows the data distribution as marks along the X-axis of the plot. The areas where most data points are concentrated are darker than the remaining portions. (b & c): A horizontal step graph showing the FWHM values in the fingerprint region (1800-800 cm^-1^) of the identified peaks for control (C) and laccase treated (T) a) finger millet straw(FM) b) paddy straw (PS). The FWHM values of each peak representing the data point are connected by a line. The horizontal connection step of the plot creates a right angle between the data points. The vertical portion of the graph represents changes in the magnitude of the data at irregular intervals

**Fig S1 a**


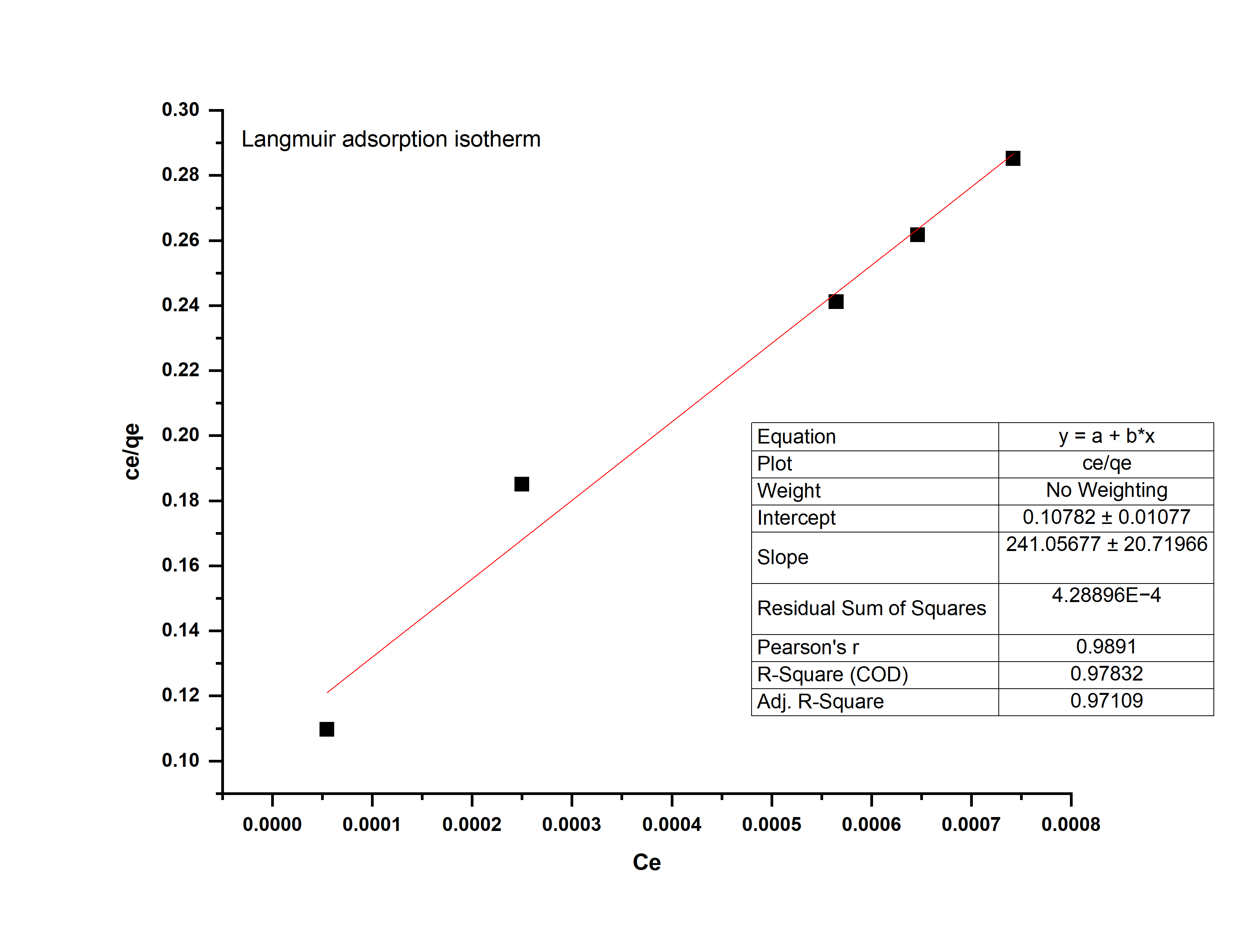


|  |  | **Value** | **Standard Error** | **t-Value** | **Prob>\|t\|** |  |  |  |  |  |  |
| --- | --- | --- | --- | --- | --- | --- | --- | --- | --- | --- | --- |
| **ce/qe** | **Intercept** | **0.10782** | **0.01077** | **10.00947** | **0.00212** | **Intercept** | **Slope** | **qmax (mg/g)** | **K_L_** | **R_L_** | **R^2^** |
|  | **Slope** | **241.05677** | **20.71966** | **11.63421** | **0.00136** | **0.10782** | **241.06** | **9.2747** | **0.0385** | **2.1543** | **0.9711** |
| **Slope is significantly different from zero  Standard Error was scaled with square root of reduced Chi-sqr.** | | | | | |  |  |  |  |  |  |

*Parameters*

**Fig S1 b**


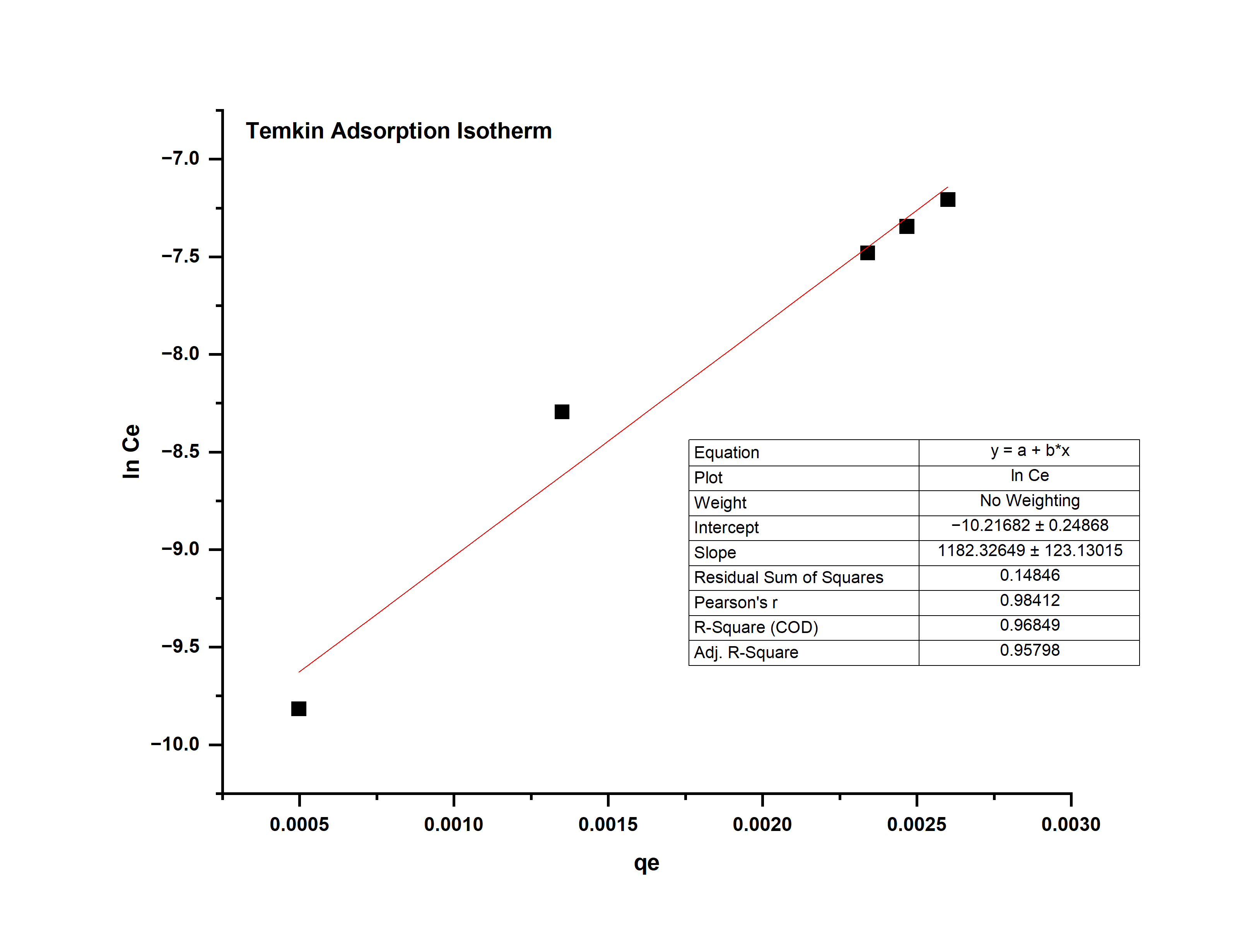


***Parameters***

|  |  | **Value** | **Standard Error** | **t-Value** | **Prob>\|t\|** |
| --- | --- | --- | --- | --- | --- |
| **ln Ce** | **Intercept** | **-10.21682** | **0.24868** | **-41.08426** | **3.17336E-5** |
|  | **Slope** | **1182.32649** | **123.13015** | **9.60225** | **0.0024** |
| **Slope is significantly different from zero  Standard Error was scaled with square root of reduced Chi-Sqr.**  **Fig S2** | | | | | |

**
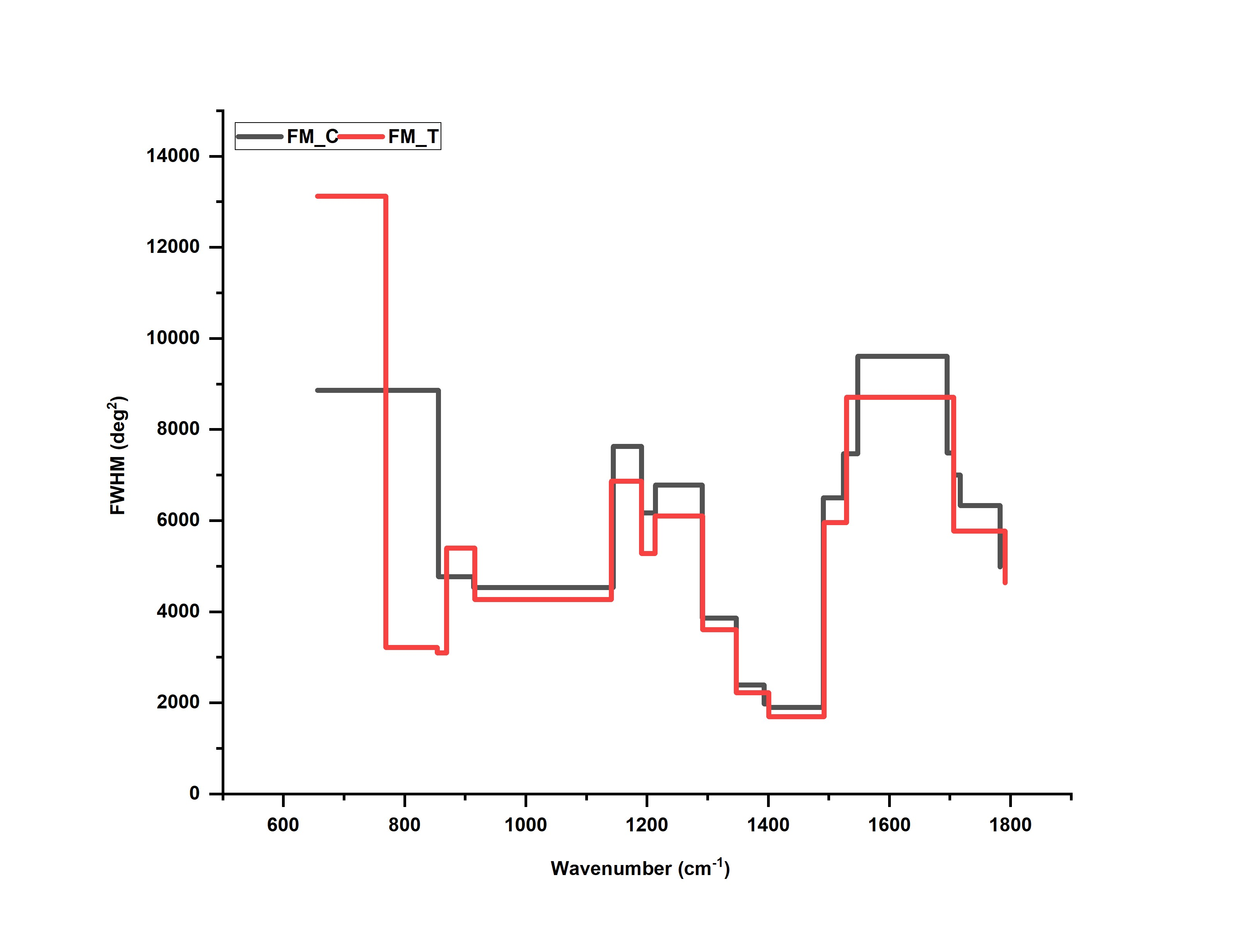
**


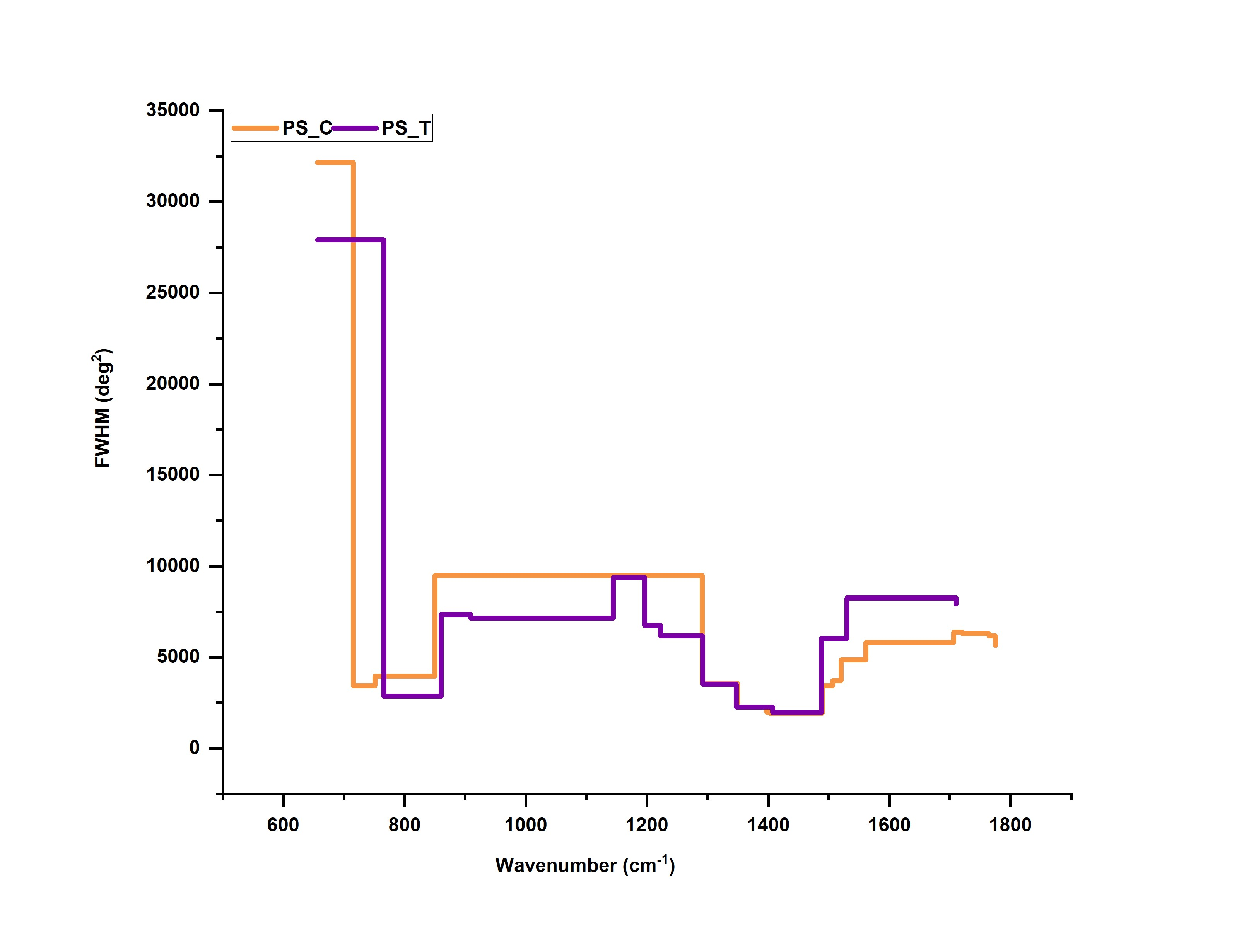

**c**

**b**

**a**
